# Trends of *Mycobacterium tuberculosis* and Rifampicin resistance in Northwest Ethiopia: Xpert® MTB/RIF assay results from 2015 to 2021

**DOI:** 10.1186/s12879-024-09135-0

**Published:** 2024-02-22

**Authors:** Sirak Biset, Milto Teferi, Haylemesikel Alamirew, Biniyam Birhanu, Awoke Dessie, Abebe Aschale, Anmaw Haymanot, Selamu Dejenie, Teshager Gebremedhin, Wondwossen Abebe, Gashaw Adane

**Affiliations:** 1 Department of Medical Microbiology, School of Biomedical and Laboratory Sciences, University of Gondar, Gondar, Ethiopia; 2 School of Biomedical and Laboratory Sciences, University of Gondar, Gondar, Ethiopia; 3 University of Gondar Comprehensive Specialized Hospital, University of Gondar, Gondar, Ethiopia; 4 Department of Immunology and Molecular Biology, School of Biomedical and Laboratory Sciences, University of Gondar, Gondar, Ethiopia

**Keywords:** Tuberculosis, Rifampicin resistance, Multi-drug resistance, Ethiopia

## Abstract

**Background:**

Tuberculosis (TB) remains one of the leading causes of morbidity and mortality worldwide, particularly in countries with limited resources. The emergence of drug resistance in *mycobacterium tuberculosis* (MTB), particularly rifampicin (RIF) resistance, hindered TB control efforts. Continuous surveillance and regular monitoring of drug-resistant TB, including rifampicin resistance (RR), are required for effective TB intervention strategies and prevention and control measures.

**Objective:**

Determine the trend of TB and RR-TB among presumptive TB patients in Northwest Ethiopia.

**Method:**

A retrospective study was conducted at the University of Gondar Comprehensive Specialized Hospital (UoG-CSH). The study included TB registration logbook data from all patients who visited the hospital and were tested for MTB using the Xpert® MTB/RIF assay between 2015 and 2021. The SPSS version 26 software was used to enter, clean, and analyze the laboratory-based data.

**Results:**

A total of 18,787 patient results were included, with 93.8% (17,615/18787) of them being successful, meaning they were not invalid, error, or aborted. About 10.5% (1846/17615) of the 17,615 results were MTB-positive, with 7.42% (137/1846) RIF resistant. Age, anti-TB treatment history, and diagnosis year were associated with the presence of MTB and RR-MTB. Tuberculosis (TB) prevalence was higher in productive age groups, whereas RR-TB prevalence was higher in the elderly. Regarding diagnosis year, the prevalence of TB and RR-TB showed a declining trend as the year progressed. While MTB was detected in 12.8% (471/3669) of new and 22.2% (151/679) of re-treatment presumptive TB patients, RR-MTB was detected in 8.5% (40/471) of new and 18.5% (28/151) of re-treatment TB cases.

**Conclusion:**

The prevalence of TB and RR-TB in the study area showed a declining trend over the years. While TB was more prevalent in productive age groups (15 to 45 years), RR-TB was more prevalent in older populations (over 45 years), than others. Moreover, patients with a history of anti-TB drug exposure were more likely to be positive for DR-TB, highlighting the need to strengthen DOT programs for proper management of TB treatment.

## Background

Tuberculosis (TB) remains one of the leading causes of morbidity and mortality worldwide, particularly in countries with limited resources [1, 2]. It is the second leading cause of death from a single infectious agent [3]. TB caused by *Mycobacterium tuberculosis* (MTB) infects about an estimated ¼ of the world population [4]. The emergence of drug-resistant (DR) MTB strains, coupled with Human Immuno-deficiency Virus (HIV) co-infection and socioeconomic factors such as poor living conditions, hampered effective TB disease control globally [5, 6]. DR-TB is responsible for roughly one-third of all antimicrobial resistance deaths worldwide, and it has devastating economic consequences, likely to cost the global economy $167 trillion between 2015 and 2050 [7, 8]. Mismanagement of TB treatment, including inappropriate anti-TB drug use or premature treatment interruption, and person-to-person transmission are the primary reasons for the continued emergence and spread of DR-TB [8–10].

Rifampicin (RIF)-resistant tuberculosis (RR-TB), defined as TB resistant to rifampicin with or without resistance to other first-line anti-TB drugs, is one of the most common types of DR-TB [11]. Since up to 90% of RR-TB cases are isoniazid resistant [12], RR-TB is often utilized as a surrogate marker for MDR-TB in countries with limited resources for MDR-TB detection [13]. Every year, an estimated half a million new MDR/RR-TB cases emerge worldwide, requiring medications that are more expensive and have more serious side effects than the first-line drugs [3, 14]. Patients with RR-TB, for example, are more likely to develop long-term physical sequelae as a result of side effects from second-line anti-TB medications [15]. Globally, 3–4% of new and 18–21% of previously treated cases are diagnosed with MDR/RR-TB in 2019 [3, 5]. In Ethiopia, however, RR-TB was reported in 1.1% of new and 7.5% of previously treated TB cases in the same year [13].

The majority of the countries with high TB, HIV-associated TB, and DR-TB burdens identified by the WHO as indicators of global action on TB for the years 2021–2025 are from Africa [3, 5], where poverty, poor-living conditions, and chronic infectious diseases are prevalent [16]. It is impossible to manage and control TB disease without continuous surveillance and regular monitoring of TB and DR-TB worldwide, which are critical for effective intervention plans and control methods. Despite reports on MDR/RR-TB in Ethiopia, data on the prevalence and trend of RR-TB in the study area is still limited. As a result, the purpose of this study was to determine the prevalence and its trend of RR-TB in northwest Ethiopia.

## Materials and methods

### Study design, area, and period

A hospital-based retrospective study was conducted at the University of Gondar Comprehensive Specialized Hospital (UoGCSH) in Gondar, Ethiopia, from January 1, 2015, to December 31, 2021. The UoGCSH provides outpatient and inpatient services for more than seven million residents in North Gondar and its surrounding areas. This hospital offers curative, rehabilitative, educational, and promotional services. It has more than 518 beds, with different health service-providing departments, including the TB clinic and laboratory. The TB laboratory receives samples from a variety of healthcare facilities for GeneXpert testing, culture, and drug susceptibility testing. The data collection period was from July to September 2022.

### Study population

We included TB registration logbook data from all presumptive TB patients tested with the Xpert® MTB/RIF assay. The 'INVALID', 'ERROR', and 'ABORTED' Xpert® MTB/RIF assay results were excluded from the inferential statistics.

### Data collection tools

We collected data from presumptive TB patients' TB registration logbooks using a structured checklist. There were six data collectors and two supervisors. Patient information, including age, sex, patient category, site of TB infection, laboratory results, and year of diagnosis, were obtained from the TB laboratory registration logbooks.

### Data quality control

We used different ways to ensure that the collected data had the required quality. For instance, we used a structured data collection checklist and regular communication with the hospital staff working at TB clinic and its laboratory. Finally, we checked the collected data and cleaned manually and entered SPSS version 26 for analysis.

### Laboratory methods

The Xpert® MTB/RIF assay, an automated in vitro diagnostic test using nested real-time PCR for the qualitative detection of MTB-complex (MTBC) and RIF resistance, was used [17]. Sputum specimens were collected in Leak-proof, sterile, screw-capped specimen collection containers: in our case, a 15 ml volume falcon tubes. If the specimen could not be processed immediately, it was transported and stored at 2–8 °C until it could. In the laboratory, the Sample Reagent, which contains sodium hydroxide and isopropanol, was poured into the sample inside the falcon tube (2:1 dilution, Sample Reagent: sample). The falcon tube was then tightly screw-capped and vigorously shaken before being incubated for 10 min. After the first incubation, the tube was shaken once more before being incubated at room temperature for another 5 min. Using a disposable transfer pipette, 2 mL of the mixture was then introduced into the labeled Xpert® MTB/RIF cartridge, which is then loaded into the Xpert® MTB/RIF instrument for DNA extraction and amplification of the *rpoB* gene. The detection consists of hybridization of the amplicon with five overlapping probes complementary to the *rpoB* “core” region determining the RIF-resistance. Results were automatically generated within 2 h and reported as ‘MTB not detected’, ‘MTB detected RR not detected’, ‘MTB detected RR detected’, or ‘MTB detected RR indeterminate’.

### Data processing and analysis

All participants’ information and laboratory data were entered and then analyzed using the SPSS version 26 (IBM Corp, Armonk, NY). Descriptive analysis was used to describe and calculate frequencies and percentages of variables. Chi-square test of proportions was used to identify a significant difference between variables, and *p*-value < 0.05 was considered as statistically significant.

## Result

### Demographic and clinical characteristics

Over the seven-year period (2015 – 2021), a total of 18,787 samples from presumptive TB patients were tested for MTB at the UOG-CSH TB laboratory. The male to female ratio was 1.34:1, with 10,742 (57.2%) patients being male. The mean age of the patients was 38.8 years (± 19.06 SD). Most of the samples were tested in 2021 (25.5%), followed by 2017 (17.4%) and 2019 (15.2%). Most of the samples came from patients between the ages of 26 and 35 (21.8%), followed by those over the age of 55 (20.3%) and between the ages of 36 and 45 (18.3%). About 19% (3582/19787) of the patients had a confirmed HIV result, whereas the rest had an unknown status (Table 1).

**Table 1:** Socio-demographic and clinical characteristics of presumptive TB patients

Variables		Frequency	Percentage
Sex	Male	10742	57.2
	Female	8045	42.8
Age in years	≤ 5	675	3.6
	6 – 15	1163	6.2
	16 – 25	3059	16.3
	26 – 35	4092	21.8
	36 – 45	3433	18.3
	46 – 55	2552	13.6
	≥ 56	3813	20.3
Year of diagnosis	2015	1838	9.8
	2016	1547	8.2
	2017	3277	17.4
	2018	1668	8.9
	2019	2863	15.2
	2020	2803	14.9
	2021	4791	25.5
Presumptive TB Type	PTB	17760	94.5
	EPTB	317	1.7
	No record	710	3.8
HIV status	Negative	2528	13.5
	Positive	1054	5.6
	Unknown	15225	81.0
Patient history	New	3990	21.2
	Re-treatment	763	4.1
	Unknown	14034	74.7
Referring unit	UoG-CSH	14753	78.5
	Health centers	1496	8.0
	No record	2538	13.5
Specimen type	Sputum	17123	91.1
	Gastric aspiration	628	3.3
	Pleural fluid	110	0.6
	Cerebrospinal fluid	96	0.5
	Peritoneal fluid	61	0.3
	Other fluids	50	0.3
	No record	719	3.8
**Total**		18787	100%

### Xpert® MTB/RIF assay results

**Fig. 1:**
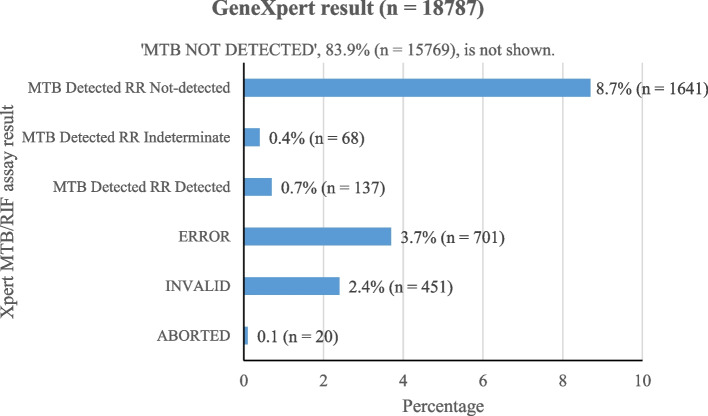
Frequency of Xpert MTB/RIF assay result at the UoGCSH TB laboratory (2015 – 2021)

Of the 18,787 samples tested, 93.8% (17,615) yielded successful results, while 6.2% (1172) did not. *Mycobacterium tuberculosis* was detected in 1846 of the successful results, with 137 and 68 being RR and rifampicin indeterminate, respectively (Fig. 1).

 In the following result presentations, we excluded patient samples with unsuccessful results for a more accurate analysis of MTB and RR-MTB prevalence.

### Prevalence of TB among presumptive TB patients

The overall prevalence of TB was 10.48% (1846/17615), with the highest prevalence observed in patients aged between 16 and 25 (17.4%), followed by 14.9% in those aged between 26 and 35 and 10.35% in those aged between 36 and 45. The highest prevalence of TB was recorded in 2015, at 23.7% (395/1670), followed by 16.2% (230/1421) in 2016 and 10.0% (275/2763) in 2017. The prevalence of TB among retreatment cases was 22.2% (151/679), which was significantly higher than the 12.8% (471/3669) among new cases. Compared to HIV negative patients, who had a TB prevalence of 11.8% (284/2407), HIV positive patients had a significantly higher prevalence of TB, at 19% (182/959) (Table 2).

**Table 2:** Prevalence of MTB among presumptive TB patients at the UoGCSH (2015 – 2021)

Variables		Frequency N^o^ (%)	MTB result		Chi-square (X^2^)	*p*-value
			Detected N^o^ (%)	Not detected N^o^ (%)		
Sex (*n* = 17615)	Male	10078 (57.2)	1060 (10.5)	9018 (89.5)	0.037	0.848
	Female	7537 (42.8)	786 (10.4)	6751 (89.6)		
Age in years (*n* = 17615)	≤ 5	633 (3.6)	39 (6.2)	594 (93.8)	414.517	< *0.001*
	6 – 15	1102 (6.3)	66 (6.0)	1036 (94.0)		
	16 – 25	2864 (16.3)	498 (17.4)	2366 (82.6)		
	26 – 35	3831 (21.8)	572 (14.9)	3259 (86.1)		
	36 – 45	3198 (18.2)	331 (10.4)	2867 (89.7)		
	46 – 55	2386 (13.6)	163 (6.8)	2223 (93.2)		
	≥ 56	3591 (20.4)	177 (4.9)	3414 (95.1)		
Year of diagnosis (*n* = 17615)	2015	1670 (9.5)	395 (23.7)	1275 (76.4)	438.148	< *0.001*
	2016	1421 (8.1)	230 (16.2)	1191 (83.8)		
	2017	2763 (15.7)	275 (10.0)	2488 (90.0)		
	2018	1595 (9.1)	132 (8.3)	1463 (91.7)		
	2019	2734 (15.5)	193 (7.1)	2541 (92.9)		
	2020	2659 (15.1)	235 (8.8)	2424 (91.2)		
	2021	4773 (27.1)	386 (8.1)	4387 (91.9)		
TB category (*n* = 4348)	New	3669 (84.4)	471 (12.8)	3198 (87.2)	41.309	< *0.001*
	Re-Rx	679 (15.6)	151 (22.2)	528 (77.8)		
HIV status (*n* = 3366)	Positive	959 (28.5)	182 (19.0)	777 (81.0)	29.633	< *0.001*
	Negative	2407 (71.5)	284 (11.8)	2123 (88.2)		
Specimen type (*n* = 16935)	Sputum	16004 (94.5)	1718 (10.7)	14286 (89.3)	14.230	< *0.001*
	Gas-aspir	624 (3.7)	39 (6.3)	585 (93.7)		
	Others	307 (1.8)	26 (8.5)	281 (91.5		
Diagnosis (*n* = 16944)	PTB	16637 (98.2)	1757 (10.6)	14880 (89.4)	1.401	0.236
	EPTB	307 (1.8)	26 (8.5)	281 (91.5)		
Refer unit (*n* = 15250)	UoGCSH	13865 (90.9)	1292 (9.3)	12573 (90.7)	103.958	< *0.001*
	HCs	1385 (9.1)	249 (18.0)	1136 (82.0)		

*MTB Mycobacterium tuberculosis*, *PTB* Pulmonary Tuberculosis, *EPTB* Extra-pulmonary tuberculosis, *UoGCSH* University of Gondar Comprehensive Specialized Hospital, *HCs* Health Centers, *Gas-asir* Gastric aspiration, *Re-Rx* retreatment

### Prevalence of RR-TB among presumptive TB patients

The overall prevalence of RR-TB was 0.78% (137/17615), with the highest prevalence observed in patients aged 16 to 25 (1.22%), followed by 1% in those aged 26 to 35. The highest prevalence was recorded in 2015, at 2.7% (45/1670), followed by 1.13% (16/1421) in 2016, and 0.76% (21/2763) in 2017. Rifampicin-resistant TB was found in 1.1% (40/3669) of new and 4.1% (28/679) of retreatment presumptive TB patients. Patients referred from nearby health centers had a higher prevalence of RR-TB, 1.6% (22/1385), than those who visited the UoG-CSH directly, 0.63% (87/13865) (Tables 2 and 3).

**Table 3:** Prevalence of RR-TB among MTB positive patients at the UoGCSH (2015 – 2021)

Variables		Frequency N^o^ (%)	Rifampicin resistance		Chi-square (X^2^)	*p*-value
			Detected N^o^ (%)	Not detected N^o^ (%)		
Sex (*n* = 1846)	Male	1060 (57.4)	73 (6.9)	987 (93.1)	1.036	0.309
	Female	786 (42.6)	64 (8.1)	722 (91.9)		
Age in years (*n* = 1846)	≤ 5	39 (2.1)	0	39 (100)	14.551	*0.024*
	6 – 15	66 (3.6)	4 (6.1)	62 (93.9)		
	16 – 25	498 (27.0)	35 (7.0)	463 (93.0)		
	26 – 35	572 (31.0)	38 (6.6)	534 (93.4)		
	36 – 45	331 (17.9)	24 (7.3)	307 (92.7)		
	46 – 55	163 (8.8)	23 (14.1)	140 (85.9)		
	≥ 56	177 (9.6)	13 (7.3)	164 (92.7)		
Year of diagnosis (*n* = 1846)	2015	395 (21.4)	45 (11.4)	350 (88.6)	18.287	*0.006*
	2016	230 (12.5)	16 (7.0)	214 (93.0)		
	2017	275 (14.9)	21 (7.6)	254 (92.4)		
	2018	132 (7.2)	8 (6.1)	124 (93.9)		
	2019	193 (10.5)	18 (9.3)	175 (90.7)		
	2020	235 (12.7)	14 (6.0)	221 (94.0)		
	2021	386 (20.9)	15 (3.9)	371 (96.1)		
TB category (*n* = 622)	New	471 (75.7)	40 (8.5)	431 (91.5)	11.862	< *0.001*
	Re-Rx	151 (24.3)	28 (18.5)	123 (81.5)		
HIV status (*n* = 466)	Positive	182 (39.0)	11 (6.0)	171 (94.0)	1.469	0.226
	Negative	284 (61.0)	26 (9.2)	258 (90.8)		
Specimen type (*n* = 1783)	Sputum	1718 (96.4)	131 (7.6)	1587 (92.4)	3.214	0.200
	Gas-aspir	39 (2.2)	0	39 (100)		
	Others	26 (1.5)	2 (7.7)	24 (92.3)		
Diagnosis (*n* = 1783)	PTB	1757 (98.5)	131 (7.5)	1626 (92.5)	0.002	0.964
	EPTB	26 (1.5)	2 (7.7)	24 (92.3)		
Refer-unit (*n* = 1541)	UoGCSH	1292 (83.8)	87 (6.7)	1205 (93.3)	1.403	0.236
	HCs	249 (16.2)	22 (8.8)	227 (91.2)		

*PTB* Pulmonary Tuberculosis, *EPTB* Extra-pulmonary tuberculosis, *UoGCSH* University of Gondar Comprehensive Specialized Hospital, *HCs* Health Centers, *Gas-asir* Gastric aspiration, *Re-Rx* retreatment

### Prevalence of RR-TB among MTB positive patients

The overall prevalence of RR-TB among MTB positive patients was 7.42% (137/1846) (95% CI = 6.2–8.7), with the highest prevalence observed in patients aged between 46 and 55 (14.1%), followed by 7.34% in those aged above 55 and 7.25% in those aged between 36 and 45. Female patients with MTB were more likely to be positive for RR-TB, 8.14% (64/786), than male patients, 6.9% (73/1060). The highest prevalence of RR-TB was 11.4% (45/395) in 2015, followed by 9.3% (18/193) in 2019 and 7.6% (21/275) in 2017. RIF-resistance was detected in 8.5% (40/471) of new and 18.5% (28/151) of re-treatment TB cases (Table 3).

### Trends of TB and RR-TB prevalence by years

**Fig. 2:**
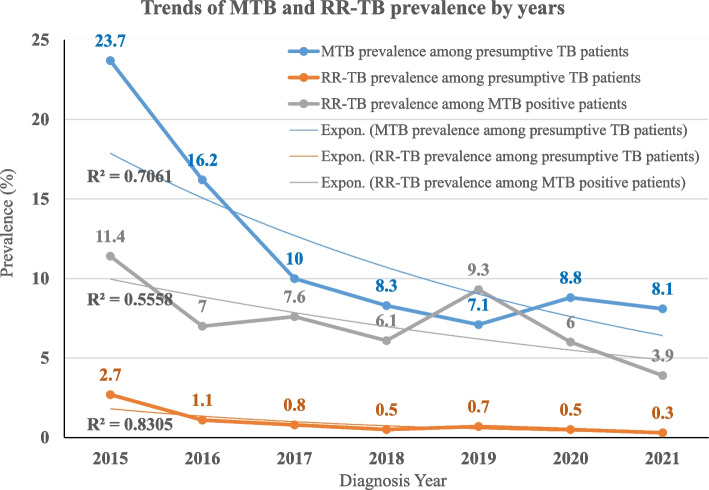
Year trend of TB and RR-TB at the UoGCSH TB laboratory (2015 – 2021)

Over the seven-year period, TB prevalence ranged from 7.1% (193/2734) in 2019 to 23.7% (395/1670) in 2015, with a significant decline as the year progressed. There was also a significant drop in prevalence of RR-TB in presumptive TB patients, which ranged from 0.3% (15/4773) in 2021 to 2.7% (45/1670) in 2015. The prevalence of RR-TB among MTB-positive patients fluctuated during a seven-year period, however, there was evidence of an overall decreasing trend; for instance, the prevalence ranged between 3.9% (15/386) in 2021 and 11.4% (45/295) in 2015 (Fig. 2).

### Associated factors.

The chi-square test was used to assess associated factors for the presence of MTB and RR-MTB in patient samples. Patient age, diagnosis year, patient TB history, HIV status, specimen type, and referring unit were all associated with the presence of MTB: in all cases, the *p*-value was < 0.001 (Table 2). Patient age (*p*-value = 0.024), diagnosis year (*p*-value = 0.006), and patient TB history (*p*-value = 0.001) were factors associated with the presence of RR-MTB (Table 3).

## Discussion

Continuous surveillance and regular monitoring of DR-TB are essential for disease management and control programs; thus, local epidemiological data on MTB and RR-MTB is critical to designing effective intervention plans and methods to control TB disease in the study area. The overall prevalence of TB in the current study was 10.5% (95% CI = 10.0 – 10.9), which is lower than previous reports from the same study area (Gondar), 24.6% [18], Adigrat, 24.3% [19], Gambella, 20.0% [20], Addis Ababa, 17% [21], Addis Ababa, 15.1% [22], Gedeo Zone, 11.8% [23], and Eastern Amhara, 11% [24]. However, it was higher than other previous reports, including Addis Ababa, 13.5% [25], Jimma, 9.3% [26], Motta, 8.4% [27], and Tigray, 7.9% [28]. Variations in the study period, sample size, geographic locations, TB epidemiology, and TB prevention and control practices could explain the disparity in prevalence. For example, prior to 2015, the Xpert® MTB/RIF assay was recommended for patients with higher risks, whereas this study included data from 2015 to 2021, when the assay was used for all presumptive TB patients.

The prevalence of RR-MTB in this study was 7.42% (95% CI = 6.2–8.7), which agrees with previous reports from Adigrat (7.12%) [19] and Eastern Amhara (8.3%) [24]. It is, however, lower than reports from Gondar (15.8%) [18], Addis Ababa [21, 22, 25], Tigray [28], and a review article in Ethiopia (9.75%) [29]. On the other hand, this prevalence is higher than reports from another parts of Ethiopia [20, 23, 26, 27]. Disparities in prevalence may be attributable to variations in study setting, study period, sample size, TB epidemiology (organization of the TB care system, features of TB treatment process, healthcare and social security legislation), study participants, and MDR-TB monitoring and control practices [30].

In this study, the prevalence of MTB was nearly equal among female (10.4%) and male (10.5%) patients, whereas RR-TB was higher among females (8.1%) than males (6.9%), though this difference was not statistically significant. A previous study (2013–2015) from the same study area reported higher MTB prevalence with nearly equal distribution in male (24.1%) and female (25.3%) patients; however, the study found a higher RR-TB in males (17.3%) than females (13.8%) [18], which is supported by a report from Gambella [20]. On the other hand, retrospective studies from Addis Ababa reported a higher RR-TB prevalence in female than male patients [21, 25], which is consistent with our findings. Despite the fact that males are at a higher risk of TB [31, 32], a report based on data from 106 countries indicated that the risk of MDR/RR-TB among TB patients is the same for males and females [33].

In the present study, MTB prevalence was significantly higher in those aged 18 to 45 years compared to younger and older populations (*p* < 0.001). This is consistent with previous reports from Ethiopia, where adults were more affected than other populations [20, 26, 28]. According to the WHO reports, TB primarily affects people in economically productive age groups, with approximately two-thirds of TB cases occurring among those aged 15 to 59 years [3, 5]. This could be related to the fact that people in this age group are more likely to get TB due to their increased exposure to the outside environment and wide range of mobility [34]. The high TB prevalence among productive age groups suggests that TB is circulating in the community, underlining the need for additional and coordinated efforts to address the problem. In another finding, RR-TB prevalence was significantly higher in TB patients aged 46 to 55 years compared to others, which is consistent with what was reported in a study from Nigeria [35]. However, study reports from Ethiopia [36–38] and elsewhere [39] do not support this finding: they reported that elderly peoples are less likely to be positive for MDR/RR-TB.

The prevalence of TB in this study was significantly higher among previously treated patients than new cases (*p* = 0.001), which is in agreement with other reports from Ethiopia [23–26, 28] and elsewhere [35–38]. According to this study, RR-MTB in new and retreatment cases was 8.5% and 18.5%, respectively, which is much higher than the national report [13]. An 8.5% prevalence of RR-MTB in new cases is also higher than the global WHO report [3, 5]. However, there are studies in Ethiopia that report almost similar findings to the current study [21, 22, 25]. According to a systematic review and meta-analysis of 16 articles in Ethiopia, DR-TB is significantly higher in previously treated TB patients than in newly diagnosed TB patients [40]. Furthermore, history of anti-TB therapy and previous TB disease are also reported as risk factors for MDR/RR-TB worldwide [41]. Drug-resistant TB is usually the outcome of prior anti-TB drug exposure, where the growth of drug-susceptible TB bacilli is inhibited whereas pre-existing DR mutants multiply freely [42]. Furthermore, mismanagement of TB treatment by healthcare workers, such as lack of treatment supervision and prescribing inappropriate regimens, combined with patient characteristics such as low literacy levels and delays in seeking healthcare, can result in poor treatment adherence, which has an impact on DR-TB prevalence [38, 43]. The higher MTB and RR-MTB prevalence in the previously treated patients highlights the need of strengthening DOT programs to properly manage TB treatment in the study area.

This study also reported that TB prevalence was higher among people with HIV (*p* < 0.001) than those naïve to it. HIV, on the other hand, was not associated with the presence of RR-MTB. This is in agreement with other study reports in Ethiopia [23, 26, 28] According to the yearly WHO report, HIV is one of the major risk factors for TB, particularly for new cases [3, 5]. HIV infection weakens immune system functions, allowing latent TB infection to progress to active TB disease. HIV infection reduces the CD4 count, impairs the function of TB-specific T-cells, induces innate immune defects, and limits the ability of macrophages to restrict TB-bacilli growth [44, 45].

This study also attempted to look at MTB and RR-MTB trends over the study periods. MTB prevalence fell from 23.7% in 2015 to 7.1% in 2019, then slightly increased to 8.8% in 2020 and 8.1% in 2021. Overall, the prevalence of MTB showed a significant decline as the year progressed, for instance, patients tested prior to 2018 had significantly higher prevalence than those tested from 2018 to 2021 (*p* < 0.001). This is consistent with reports from Addis Ababa [25]. However, other reports from Ethiopia showed a fluctuated trend [21, 23]. The prevalence of RR-TB among MDR-positive patients in the study area, on the other hand, fluctuated between 3.9% in 2021 and 11.4% in 2015. The use of the Xpert® MTB/RIF assay in Ethiopia for selected patients with higher risks may have contributed to the increased prevalence in 2015. However, as the assay became recommended for all TB presumptive patients in the following years, the number of patients diagnosed increased significantly over time, reducing the proportion of positive cases.

The strengths of this study include: the data is from a hospital that serves over 7 million people, which could help point out possible gaps related to TB interventions in the area; the study findings are based on a large sample size, which enhances their representativeness; and showing prevalence trends over 7 years could assist in evaluating the quality of TB services in the area. While our study has these strengths, we recognize that it has some limitations. Since we used secondary data, it was impossible to access the full range of factors for RR-TB. For instance, patient data on sociodemographic, behavioral, and clinical factors were not fully recorded in TB laboratory result registration books. Furthermore, there were a considerable number of patients with ‘unknown’ status of variables or factors, which could affect the possible association that the variables may have with MTB and RR-MTB prevalence.

## Conclusion

The overall prevalence of TB and RR-TB in the study area showed a decline trend over the years. However, TB is still more prevalent in productive age groups, underlining the need for additional and coordinated efforts to address the problem in these populations. Furthermore, a relatively high overall incidence of RR-TB among new TB cases in the study area may indicate that DR-TB is circulating in the community, demanding enhanced early DR-TB detection as well as strengthening TB infection prevention and control measures. Moreover, a higher prevalence of RR-TB among elderly individuals and those with a history of anti-TB drug exposure also emphasizes the need of strengthening DOT programs for proper management of TB treatment.

## Data Availability

All data supporting the findings of this study are available within this paper.

## Abbreviations

CI: Confidence Interval
DOT: Directly Observed Therapy
DR: Drug-resistance
DR-TB: Drug-resistant Tuberculosis
HIV: Human Immunodeficiency Virus
MDR: Multidrug resistance
MDR-TB: Multidrug-resistant Tuberculosis
MTB: *Mycobacterium tuberculosis*
RIF: Rifampicin
RR: Rifampicin resistance
RR-MTB: Rifampicin-resistant *Mycobacterium tuberculosis*
RR-TB: Rifampicin-resistant Tuberculosis
SD: Standard deviation
SPSS: Statistical Package for Social Sciences
TB: Tuberculosis
UoG-CSH: University of Gondar Comprehensive Specialized Hospital
WHO: World Health Organization
